# Spatio-temporal distribution of negative emotions on Twitter during floods in Chennai, India, in 2015: a post hoc analysis

**DOI:** 10.1186/s12942-020-00214-4

**Published:** 2020-05-28

**Authors:** Dhivya Karmegam, Bagavandas Mappillairaju

**Affiliations:** 1 School of Public Health, SRM Institute of Science and Technology, Chennai, Tamil Nadu 603203 India; 2 Centre for Statistics, SRM Institute of Science and Technology, Chennai, Tamil Nadu 603203 India

**Keywords:** Emotional analysis, Spatial statistics, Disaster mental health, Geographic information system, Twitter

## Abstract

**Background:**

Natural disasters are known to take their psychological toll immediately, and over the long term, on those living through them. Messages posted on Twitter provide an insight into the state of mind of citizens affected by such disasters and provide useful data on the emotional impact on groups of people. In 2015, Chennai, the capital city of Tamil Nadu state in southern India, experienced unprecedented flooding, which subsequently triggered economic losses and had considerable psychological impact on citizens. The objectives of this study are to (i) mine posts to Twitter to extract negative emotions of those posting tweets before, during and after the floods; (ii) examine the spatial and temporal variations of negative emotions across Chennai city via tweets; and (iii) analyse associations in the posts between the emotions observed before, during and after the disaster.

**Methods:**

Using Twitter’s application programming interface, tweets posted at the time of floods were aggregated for detailed categorisation and analysis. The different emotions were extracted and classified by using the National Research Council emotion lexicon. Both an analysis of variance (ANOVA) and mixed-effect analysis were performed to assess the temporal variations in negative emotion rates. Global and local Moran’s I statistic were used to understand the spatial distribution and clusters of negative emotions across the Chennai region. Spatial regression was used to analyse over time the association in negative emotion rates from the tweets.

**Results:**

In the 5696 tweets analysed around the time of the floods, negative emotions were in evidence 17.02% before, 29.45% during and 11.39% after the floods. The rates of negative emotions showed significant variation between tweets sent before, during and after the disaster. Negative emotions were highest at the time of disaster’s peak and reduced considerably post disaster in all wards of Chennai. Spatial clusters of wards with high negative emotion rates were identified.

**Conclusions:**

Spatial analysis of emotions expressed on Twitter during disasters helps to identify geographic areas with high negative emotions and areas needing immediate emotional support. Analysing emotions temporally provides insight into early identification of mental health issues, and their consequences, for those affected by disasters.

## Introduction

The world has witnessed a rise in natural disasters such as floods, hurricanes and storms due to the effects of climate change [1]. During many of these events, populations affected by natural disasters are susceptible to a wide range of mental health problems [2, 3]. Consequently, a number of people are found to experience post-traumatic stress disorders, anxiety, depressive disorder, and distress as a direct result of living through the event [4–7]. Identifying the psychological consequences of disasters is a challenge for researchers, and traditional data collection methods such as questionnaires, interviews, and focus groups discussions may introduce delays and a time lag. Further, timely capture of data immediately before, during, and after a disaster may be impossible to accomplish and is likely to be lost [8]. During disasters, people are known to convey messages for help and express their feelings through social media. Facebook status updates, tweets, and YouTube videos posted during the disaster provide an insight into the prevailing mood of people in specific locations [9–11]. During the past decade, social media has been employed by disaster management teams to study situational awareness [12–14], plan response [15], and undertake relief activities [16–18]. Social media updates during disasters provide real-time data that is not possible to be collected using traditional data collection methods. In particular, real-time data can be tapped effectively to identify the mental health status of individuals or an entire population [19–22]. Twitter has proved effective for such an analysis. Using emotional analysis, negative emotions expressed in tweets can be classified into fear, anger or sadness and tied to different disaster time periods [23, 24]. Further, a spatio-temporal analysis of tweets can be done to identify the spatial clusters that display negative emotions at specific points in time [25, 26]. Negative emotions are particularly important as they have been shown to result in adverse mental health consequences in individuals, especially in a disaster context [2, 27].

Studies have identified people experiencing psychological consequences of traumatic events from their social media updates by extracting emotions and sentiments expressed by them from those updates [23, 28–32]. Some of the studies have also investigated the geographic variations in emotions [25, 26, 33]. The identification of emotions of the distressed population by location provides an insight into the stress experienced by people in specific locations. Analysis of the general mood and alertness levels of a population cluster-wise also becomes possible [34]. The emotional status of people in a spatial cluster can be further drilled down to specific time periods using a temporal analysis. Once real-time spatio-temporal information on specific clusters experiencing severe stress becomes available, public health authorities can make early interventions to arrest further damage and, where required, also provide appropriate mental health interventions [35, 36].

The psychological status of people before a disaster acts as a predictor of psychological problems they experience during a disaster [27], which in turn gives an idea about the psychological stress that they encounter after the disaster. A spatio-temporal analysis to identify the clusters in which people display negative emotions, which could quickly deteriorate into more severe psychological problems, thus becomes important. Similarly, spatio-temporal analysis of clusters during a disease outbreak has helped in the identification of hotspots, areas of outbreak and the population at risk [37–39]. This data has helped the concerned management teams to undertake emergency public health interventions [40, 41]. If data on negative emotions of people can be gathered along with their time stamps, in addition to their geographic location, it provides an insight into the spatial clusters that experience extreme negative emotions and the specific time of such occurrence. To the best of our knowledge, no study has undertaken a spatio-temporal analysis of negative emotions using data from social media in India [42]. Using geo-coded Twitter messages posted around the time of floods during November/December 2015 in Chennai, the southern Indian city and capital of Tamil Nadu state, we undertook a spatio-temporal study of negative emotions expressed by people in the affected areas. The aim of our study was three-fold: (i) quantify emotions (extracted from Twitter feed) expressed by the people of Chennai before, during and after the 2015 floods; (ii) understand the spatial and temporal variations of negative motions across Chennai city via tweets at the ward level; and (iii) compare the negative emotion rates before and during the floods with those during and after the floods.

## Methods

The objective of this study was to gain an understanding of the spatial and temporal variations of negative emotions across Chennai during an unexpected disaster. To ascertain the temporal variations in negative emotion rates, tweets posted by people in Chennai before, during and after the floods that ravaged the city in November/December 2015 were used in our study.

### Study setting

**Fig. 1:**
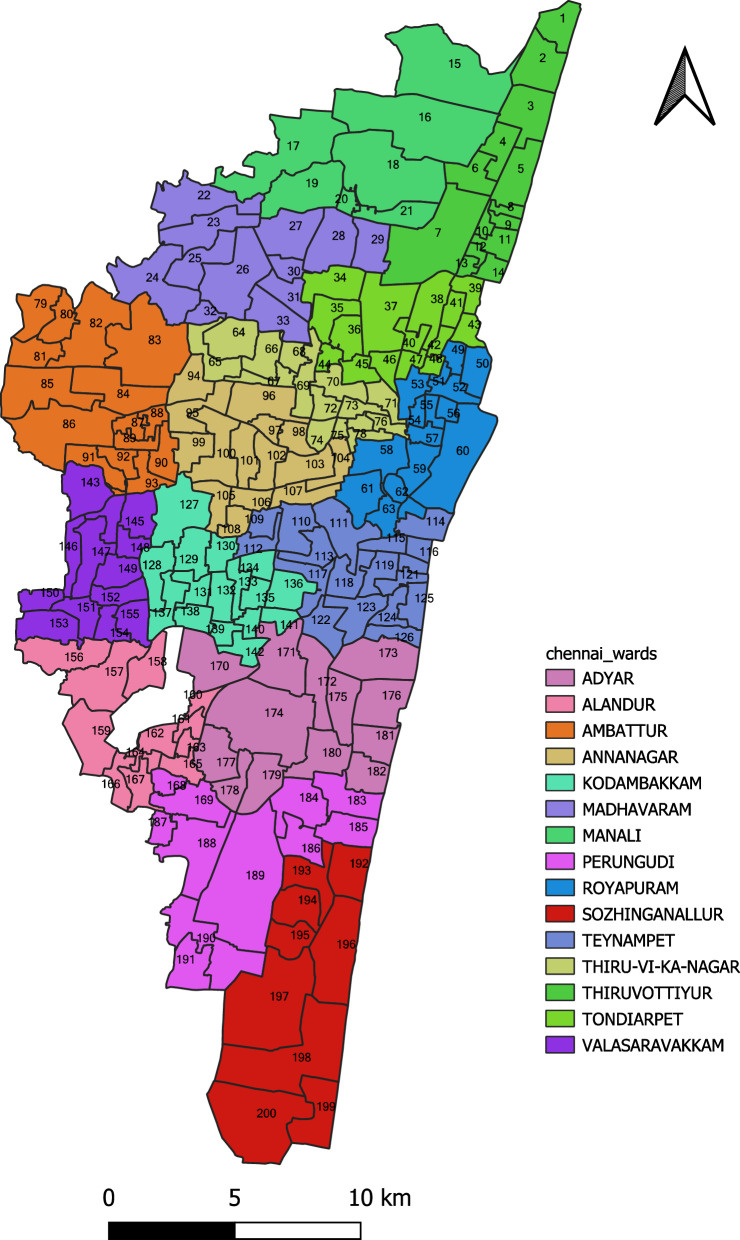
Study area, Chennai city (ward numbers and zone names displayed)

The Corporation of Chennai has divided the city’s metropolitan area into 15 zones. These zones are further subdivided into 200 wards for administrative convenience. Figure 1 shows a map of Chennai that identifies all the zones and wards. Before discussing the study method, it would be pertinent to describe the dire situation the city was put in due to the floods in November/December 2015.

Due to its flat topography, Chennai already faces the problem of poor drainage capacity in some areas. The city has been experiencing severe floods once in a decade from the 1970s. Heavy rainfall and the resultant flooding threw normal life out of gear and affected the city’s population in various ways in 1976, 1985, 1996, 2005 and 2015 [43]. In the last episode of severe floods in 2015, the city was battered beyond its limits as the Meteorological Department reported that the rainfall was in far excessive levels than that during the seasonal monsoon the city experiences between October and December every year [44]. The city had witnessed one episode of heavy rainfall in late November, which already caused flooding in low-lying areas and had filled several small and large water bodies.

One of the reservoirs providing water supply, the *Chembarambakkam* Lake, was almost full when heavy rains lashed the city in early December. As the incessant rains began to breach the reservoir, several thousands of cusecs of water were released into the *Adyar* River flowing through the city, which compounded the situation [43]. As water levels surged in the river, the areas that were a few kilometres from the river bank were also heavily flooded, in addition to those near the bank, which bore the brunt of unexpectedly high flooding. Water levels rose as much as 6 to 8 metres in the thickly populated residential areas. The residents, caught unawares by the surging waters that rose up to the first floor of their homes, and in some cases even up to the second floor, were put into excruciating hardship and some took shelter in the terrace for as long as three days in extreme cases [45]. The state government said that 1.8 million people were moved from low-lying areas to relief camps as a precautionary measure before the floods. But flooding rose to unimaginable levels in several areas that were far away from large water bodies. Media estimated the death toll at approximately 500 peoples and economic losses were put at approximately INR 200 million [46]. Several people lost their homes, valuables and belongings cutting across socio-economic divisions. Naturally, the psychological impact of the floods on the affected population was pretty harsh [43, 47].

### Methodology

#### Data collection

As Twitter allows sharing of data through its application programming interface (API), we harvested geo-coded tweets posted by the people of Chennai in two languages, English and Tamil. Retweets were excluded. Based on media reports, the duration of disaster was fixed between 21 November and 10 December. As the study spanned time before and after the disaster as well, the tweets posted between 1 November 2015 and 30 December 2015 were collected restricting the area to 25 miles around Chennai’s geographical coordinates, 13.0827°N, 80.2707°E, for studying the spatial and temporal variations in negative emotion rates. Three distinct periods for this study were identified: pre-disaster (1 November to 20 November), disaster (21 November to 10 December) and post-disaster (11 December to 30 December).

#### Classification of emotions

The tweets that were taken up for the study were subjected to pre-processing before further analysis. First, tweets in Tamil were translated to English. Then, duplicate tweets, user mentions and external links were removed from the tweets. The word-emotion association lexicon of the National Research Council (NRC) sentiment and emotion lexicons was used to classify the emotions from the final tweets chosen for our study [48]. The NRC Sentiment and Emotion lexicons is a collection of seven lexicons, but the widely used is the word–emotion association lexicon. In this lexicon, a collection of words in English is associated with basic emotions and sentiments. The NRC lexicon has found application in earlier research for the classification of emotions in different contexts and varied domains [35, 41, 49, 50]. The emotional classification of tweets in this study was done using a software package—the Syuzhet package in R [51]. This package uses its four sentiment lexicons, built into it, to extract and plot sentiments from textual data. Scores are assigned to each tweet based on emotional terms present in it and eight emotions (anger, anticipation, disgust, fear, joy, sadness, surprise and trust) are used to score the tweets. Among these emotions, four emotions—anger, disgust, fear and sadness—are considered to be negative emotions and the tweets containing one of these emotions are coded as 1. Tweets that have no negative emotion are coded 0.

Once the negative emotions were identified from the tweets, they were then temporally classified into three categories: before, during and after the disaster. Spatial identification of the tweets was done by plotting the geo-coordinates of the tweet over the ward-level map of Chennai using Quantum GIS (QGIS), a free and open-source information system used to analyse spatial data. Tweets that lie outside the city’s map were removed from our analysis and wards that didn’t feature any negative tweet in any of the three periods of our study were not considered for further analysis. We then calculated the rate of negative emotions (i.e. number of tweets expressing negative emotions) at the ward level. When we found only a few instances of negative tweets in some wards, spatial empirical Bayes smoothing [52] was applied to handle variance instability for calculating the rate of negative tweets. Spatial neighbours were identified using queen contiguity weights matrix. The open-source software GeoDA, employed for spatial data exploration, analysis and modelling, was used to calculate the negative emotion rates during the three time periods of our study—before, during and after the disaster. Thus these calculations provided the rates of negative emotions across the three time periods in different wards of Chennai.

#### Spatial and temporal analysis

Before undertaking the spatial analysis of the tweets, we used analysis of variance (ANOVA) followed by multiple pairwise comparisons to understand if rates of negative emotions show any significance as a whole and also if there is any significance in the rates of emotions such as anger, disgust, fear and sadness across all time periods. Pairwise comparison was done using Tukey honest significant difference (Tukey HSD) test. We then undertook a longitudinal analysis using mixed effect model (also known as mixed error-component model) to ascertain the temporal evolution of negative emotions across the time periods around the disaster. Mixed model is an extension of simple linear regression model and it has the capability to deal with both fixed and random effects simultaneously. This model in employed in contexts where there exist no independent data but only a time-dependent panel data. In our case, negative emotions rates across the three time periods were treated as the panel data [53].

Global Moran’s I and local Moran’s I statistics were applied to measure spatial autocorrelation, spatial distribution and significant clusters of negative emotions in our study area. We used the spatial regression model to compare the rates of negative emotion rates between the three time periods. We chose spatial regression model because we found autocorrelation of residuals while performing classic regression. Based on log-likelihood and Akaike info criterion (AIC), the spatial lag model was chosen for performing regression on the rates of negative emotions before, during and after the disaster period.

We also undertook a thematic analysis of the tweets manually to gain an insight into the concerns of citizens that triggered their negative emotions during the disaster.

## Results

### Classification of negative emotions and their rates

Our search criteria yielded a total of 5696 original geo-coded tweets (excluding retweets and duplicates) within Chennai area in English and Tamil. Among them, 2050 tweets appeared before the disaster, 1864 during the disaster and 1782 after the disaster. When negative emotions were extracted from these tweets and a combined index for negative emotions was made, there were 17.02% of tweets indicating negative emotions before the disaster, 29.45% expressing negative emotions during the disaster and 11.39% having negative emotions after the disaster. Table 1 shows the total number of tweets, tweets containing negative emotions and also the number of tweets that expresses anger, disgust, fear and sadness in all the considered time periods.

**Table 1:** Total number and percentage of tweets that express negative emotions

Disaster time period	Tweet count (percentage)					
	Total (*N*)	Negative emotion (combined index)	Anger	Disgust	Fear	Sad
Before disaster (1 November to 20 November 2015)	2050	349 (17.02%)	174 (8.49%)	75 (3.66%)	46 (2.24%)	196 (9.56%)
During disaster (21 November to 10 December 2015)	1864	549 (29.45%)	224 (12.04%)	174 (9.33%)	326 (17.49%)	311 (16.68%)
After disaster (11 December to 30 December 2015)	1782	203 (11.39%)	62 (11.39%)	71 (3.98%)	39 (2.19%)	135 (7.58%)

Additional information on specific location (geographical coordinates) of negative tweets and the ward from which they originated are provided in Additional file 1 for all the three time periods.

A sample of tweets containing negative emotions are provided below:

*“Am so****sad****of#rainwater because in my home out side full of#rainwaterno help for us in#Chennainear#muthukumaranbackside”*

***“Worst****@ its best of Rain in Chennai: No Electricity, No milk, no food.*${\$\$\$\$\$\$}$*Oh God please save our Chennai”*

*“At last no power, full of****fear****!#chennairains”*

**Fig. 2:**
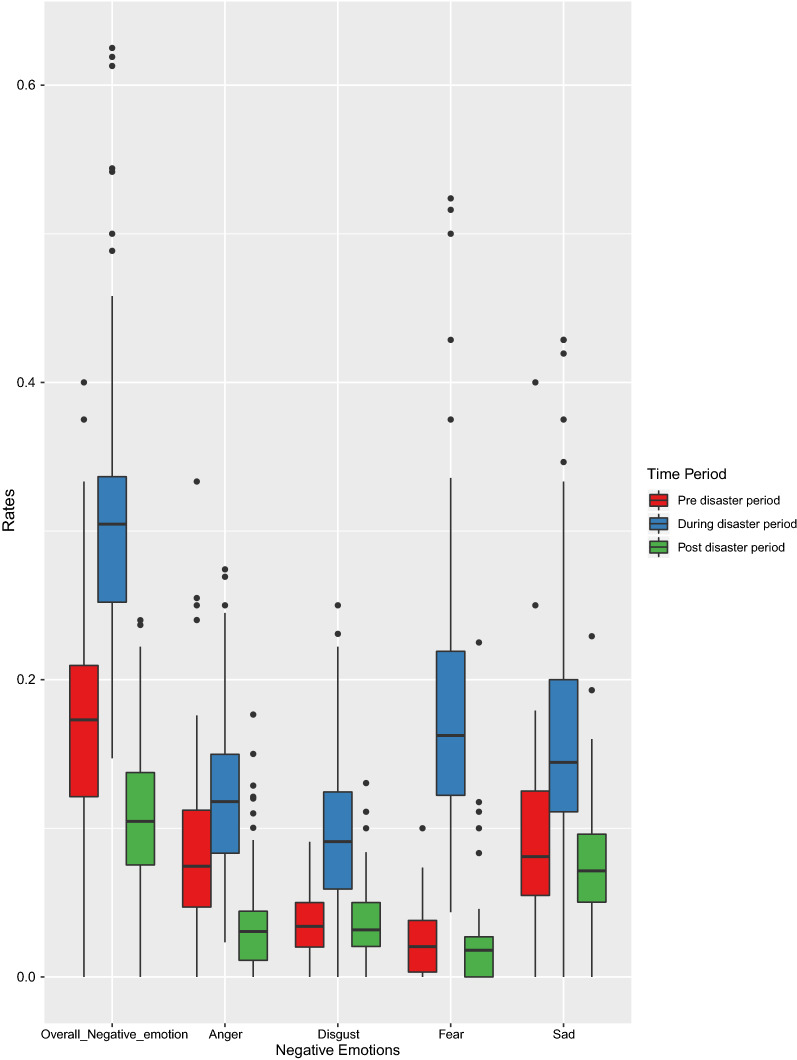
Variation of rate of negative emotions with time

As 94 wards did not exhibit any negative emotions as per our analysis, we considered only the remaining 106 wards for statistical analysis. In Additional file 2, we have provided information on the number of tweets containing negative emotions ward-wise and rates of negative tweets for all the three time periods. Figure 2 provides the comparison of the rate of negative emotions with time and it is clearly observed that negative emotions peaked during the disaster period and they cooled down considerably after the disaster.

### Temporal variation in the rate of negative emotions

Statistical analysis of tweets containing negative emotions such as anger, disgust, fear and sadness across all time periods using ANOVA and Tukey HSD showed a significant difference in the overall rate of negative emotions between the time periods. The mean difference in the rate of negative emotions before and during the disaster was 0.13912 (p < 0.05). Similarly, the rate of negative emotions during and after the disaster showed a mean difference of − 0.05828 (p < 0.05). Further, negative emotion rates varied significantly before and after the disaster (mean difference = − 0.19740, p < 0.05).

In Table 2, a statistical comparison of negative emotion rates is provided. We observe from the table that anger was the predominant emotion expressed in tweets across all the time periods. The rate of tweets expressing other three emotions—disgust, fear and sadness—varied between time periods, but there was no significant difference in their rate before and after the disaster. Therefore, we infer that the tweets expressing anger were significant in all time periods and those expressing disgust, fear and sadness peaked during the disaster but remained almost same before and after the disaster.

**Table 2:** Mean differences and statistical significance of the negative emotion rates with respect to time

Negative emotion	Time	Mean difference	Significance (99% confidence interval)
Anger	Before vs. during disaster	− 0.03836	< 0.01
	During vs. after disaster	0.08694	< 0.01
	After vs. before disaster	− 0.04859	< 0.01
Disgust	Before vs. during disaster	− 0.06516	< 0.01
	During vs. after disaster	0.06292	< 0.01
	After vs. before disaster	0.00225	0.886
Fear	Before vs. during disaster	− 0.15866	< 0.01
	During vs. after disaster	0.16104	< 0.01
	After vs. before disaster	− 0.00238	0.949
Sad	Before vs. during disaster	− 0.07404	< 0.01
	During vs. after disaster	0.08871	< 0.01
	After vs. before disaster	− 0.01468	0.193

We then studied the evolution of the rates of negative emotions (dependent variable) across time periods using a linear mixed model as the data were normally distributed. The ward number was kept as the subject variable and the various time periods were the contributing factors. The random effects model, showed the variance in negative emotion rates between wards to be 0.0013. The intercepts at the ward level were close to zero, indicating no significant deviation from the model intercept.

Table 3 shows the results of the application of fixed effect model. The negative intercept for the post-disaster period shows that the rate of negative emotions came down drastically during that period when compared to the pre-disaster and disaster periods. The rate of negative emotions was higher during the disaster than that during the pre-disaster period as evident from intercept values (showing an increase of 0.1391). These results validate the earlier statistical results of our study that the rate of negative emotions was highest during the disaster period compared to the pre-disaster and post-disaster periods, registering a significant decrease in the post-disaster period.

**Table 3:** Fixed effect model estimates (linear mixed model)

Time Period	Estimate (co-efficient)	*t*-value
(Intercept)	0.1678	22.915
During disaster	0.1391	15.249
After disaster	− 0.0583	− 6.388

### Spatio-temporal variations of negative emotions

**Fig. 3:**
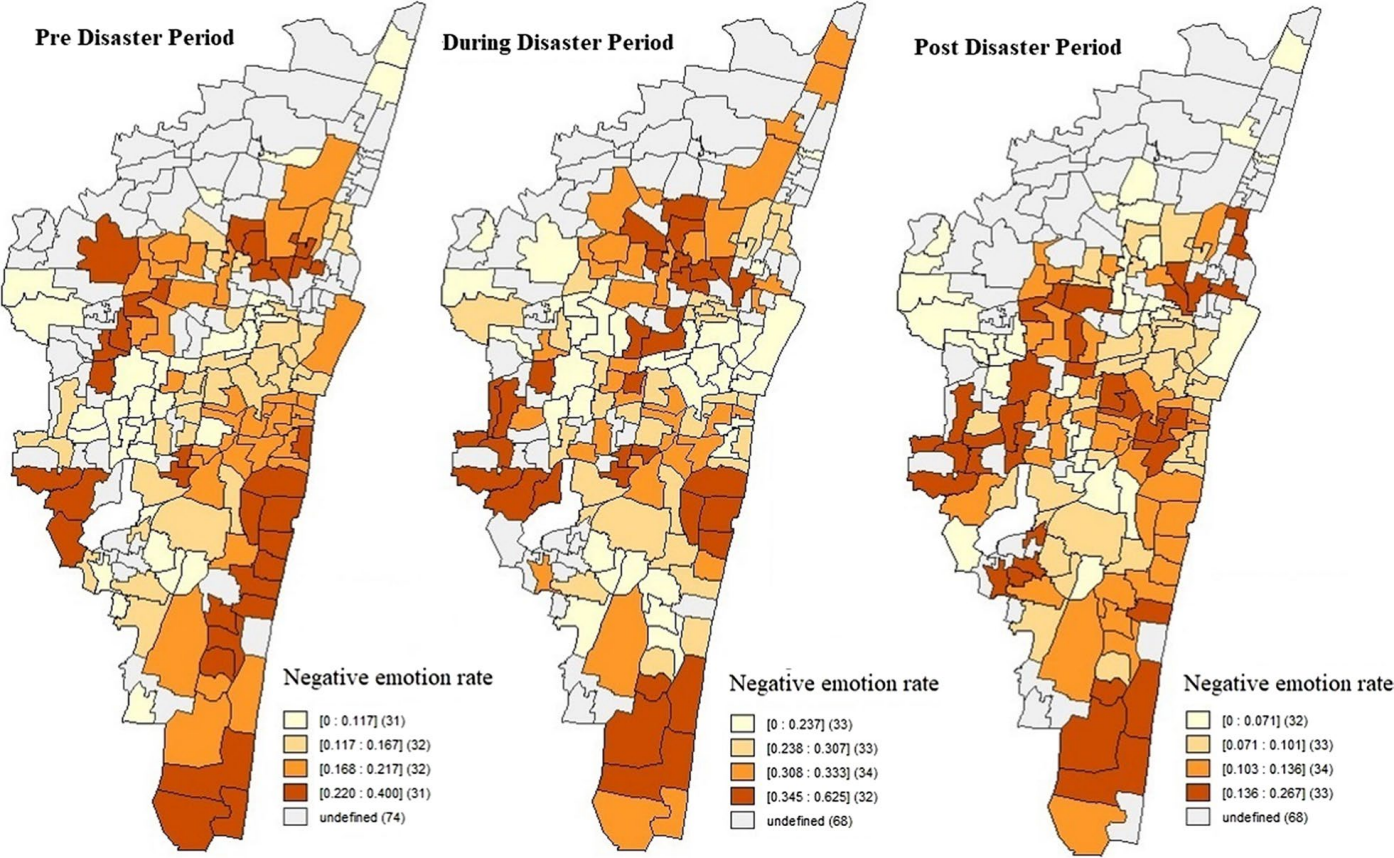
Distribution of negative emotion rates across Chennai

The negative emotion rates showed a clear variation between wards and across the studied time periods. From Fig. 3, which shows the negative emotion rates across the different wards, it can be observed that the wards 198 (in zone 15, *Shozhinganallur*) and 46 (in zone 4, *Tondiarpet*) recorded the highest rate of negative emotions across all the time periods.

**Fig. 4:**
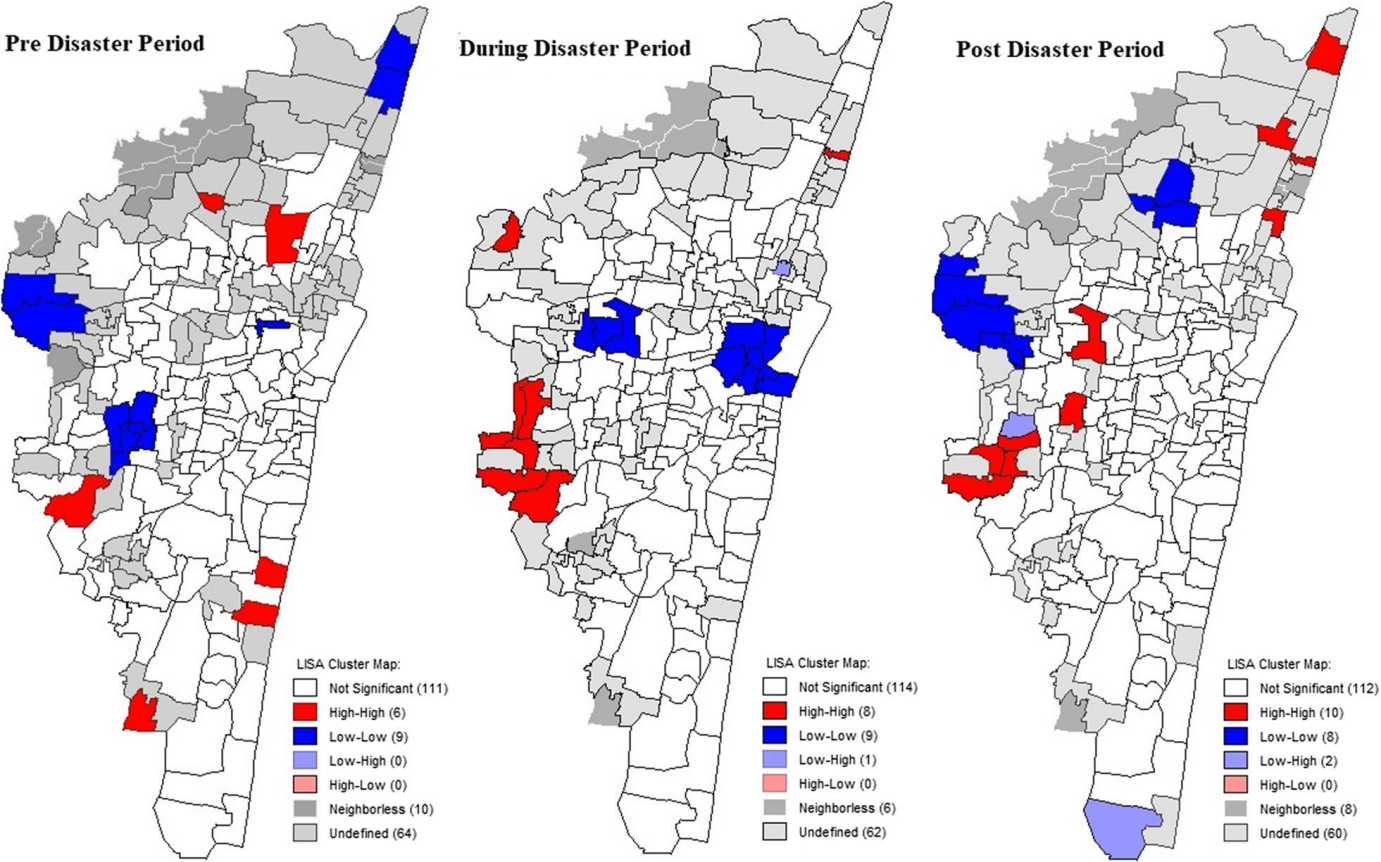
LISA cluster maps before, during and after the disaster period

To understand the spatial distribution of negative emotions, global Moran’s I values were generated. The values of 0.643 (before disaster), 0.595 (during the disaster) and 0.587 (after the disaster) indicated a positive spatial autocorrelation (similar negative emotion rates cluster together). To identify the clusters displaying negative emotions, cluster maps of negative emotion rates across all time periods from local Moran’s I values as a local indicator of spatial association (LISA) were generated (Fig. 4). From the LISA maps in the figure, it was inferred that zones 11 (*Valasaravakkam*) and 12 (*Alandur*) displayed a cluster of high level negative emotion rates. Specifically, wards 146, 147, 150, 151, 156 and 157, falling under these two zones were identified as cluster of high negative emotion rates. The high rates of negative emotions persisted in wards 151 and 156 even in the post-disaster period.

For a comparison of negative emotion rates between the time periods, simple regressions were performed. During simple regression, Moran’s I (error) value of 0.6142 (before vs. during disaster) and 0.3816 (during vs. after disaster) were obtained, showing that there exists a spatial autocorrelation among the residuals. Spatial regression indicates that there was no significant association and correlation between negative emotions before and during the disaster (*p* value = − 0.84543). In multivariate regression, there was a significant association and positive correlation (*p*-value = − 0.02273, beta = 0.09859) between negative emotion rates during and after the disaster. Pre- and post-disaster negative emotions are not associated significantly (*p*-value = 0.06242). Based on the likelihood ratio test for spatial lag dependence (*p*-value = 0.00), we found that introducing the lag term enhanced the regression model fit but did not take away the spatial effects. The results of spatial regression model are given in Additional file 3.

### Thematic analysis

To gain further insight into the key concerns of people during the disaster, we manually performed a thematic analysis of negative emotions identified from tweets posted during the disaster period (*N* = 549). The extracted topics that were of particular interest were rain and flood scenario, power, network connectivity (phone and internet) and media. We did not undertake this thematic analysis for the pre- and post-disaster periods as they were considered irrelevant.

The rain and flood scenario was the most discussed topic during the disaster. Fear and sadness were the predominant emotions displayed by people when they worried about the rain and flooding. A sample of tweets are given below:

*“ohhh God. I know. It’s scary. And they say the downpour is just going to get worse.”*

*“Really been afraid, Chennai sinks. Roads are full of water, rain encounters Chennai a lot, heavy rainfall, city can’t withstand anymore…”*

*“Oh God, pls stop this rain! :(:(#Chennai”*

The Chennai residents expressed anxiety over power and network connectivity as well, which was the second frequent topic expressed in tweets. Power and Internet connectivity were essential to receive information about the developing situation as well as keep communication alive with key contacts during the disaster. Fear was the predominant emotion inferred from these tweets. A sample of these tweets are given below:

*“no network coverage here in Chennai since morning why so? How long still like this? :*-*(“*

*“networks down! Power cut! Horrible! Save chennai! Save TN! Unstoppable rain #chennairains #ChennaiFloods”*

As many parts of Chennai were inundated and roads damaged, severe traffic snarls occurred in many junctions as well as arterial roads. People frequently expressed disgust and anger towards traffic issues. A sample of those tweets are given below:

*“Is this worst traffic recorded in Chennai ever? Stuck in OMR* - *5kms in 3* *h #chennairains #chennaitraffic”*

*“Traffic sucks #GST”*

In a few tweets, some vented their anger at the national media for not adequately paying attention to the issues related to the unprecedented Chennai floods. A couple of tweets related to this topic are as follows:

*“Shame on National media which covered Nepal Earthquake extensively and exclusively not giving that much care to #chennairains”*

*“#chennairains Why d national media is not involving.. Pretty shame on d Medias who just work for their TRPs..”*

This analysis enables us to understand the key concerns of Chennai citizens during the disaster. If this data becomes available in real time to disaster relief teams, they could act immediately to address those concerns. The context behind the expression of negative emotions such as anger, fear, sadness and disgust can be clearly understood from the tweets.

## Discussion

From our analysis, we observed that the negative frame of mind of the affected population was predominant during the 2015 floods that ravaged Chennai. As already mentioned, negative emotions ran high in tweets during the disaster compared to the pre- and post-disaster period. What was clearly visible was that the negative emotions such as fear, sadness and disgust remained almost the same between pre- and post-disaster period, spiking only during the disaster. The rate of tweets expressing anger differed significantly between pre- and post-disaster period, dropping very significantly after the disaster. If the high rate of negative emotions persisted even after the disaster, then people in the areas expressing such emotions are in need of psychological support [54].

Social media, especially tweets posted by people, have been used to assess risk or damage experienced by the people during a disaster in previous studies [55, 56]. Fear was the most expressed emotion during the floods in Chennai, and a significant increase in fear was observed during the disaster compared to the pre-disaster period. The cause of fear during the disaster was the sudden increase in water levels within a short period as a result of unexpected torrential rains that lashed the city between 30 November and 2 December 2015 [57]. Through our spatio-temporal analysis, we were able to identify the clusters that expressed high negative emotions before, during and after the disaster. Four zones—*Alandur*, *Valasaravakkam*, *Tondiarpet* and *Shozhinganallur*—were found to display high negative emotion rates during and even after the disaster. These findings from our analysis correlate with the worst-affected areas of Chennai. The wards 198 (Old Mahabalipuram Road in the *Shozhinganallur* zone) and 156 (*Manapakkam* in the *Alandur* zone) had to endure flooding as the water level rose to 5 to 6 metres and to make matters worse, these areas remained inundated for 10 to 20 days [43]. Two other zones—*Valasaravakkam* and *Tondiarpet*—had moderate flooding (water level of 0.3 to 1 m), but the problem was exacerbated by the low-income groups living in the area with high population density [58]. A very large number of people across the city encountered housing collapse to damage to vehicles to loss of valuables and belongings due to water entering homes as a result of heavy rains and flooding. After the rain stopped, residents in the worst-affected zones, as well as in several parts of the city, didn’t have access to essential commodities such as safe drinking water, milk, food and power. Prolonged inundation, economic loss and inaccessibility to basic needs contributed to people harbouring negative emotions, especially in the above mentioned areas.

Identifying and mapping emotions over geographical areas provides information on the distribution of discomfort or distress among the affected population during disasters [59, 60]. Prominent clusters displaying negative emotions were identified in the wards of *Valasaravakkam* and *Alandur* zones. Social, demographic and environmental characteristics of a location also contribute to spatial dependence in those clusters. Inundation and power cuts in the neighbouring wards may also affect the population, leading to the formation of spatial clusters displaying negative emotions. This could be true of the clusters we identified in the zones of *Alandur* and *Valasaravakkam*. These areas were flooded heavily and suddenly because they were on the banks of or in close proximity to the *Adyar* River [43] into which excessive water was discharged from the *Chembarambakkam* Lake, which is one for the reservoirs supplying water to the city. The socio-demographic characteristics such as income and household also affect the way in which people respond to unexpected disasters. Naturally, the most affected areas were those housing a population having a low socio-economic status. We strongly feel that if the cluster maps that display negative emotions rates are available real time during disasters, it would prove very useful for the disaster management teams to identify those areas and respond quickly by mobilising public health resources and also offer psychological support to the affected population. This will go a long way in avoiding the psychological fallout of a disaster.

We also found that there is an association between negative emotion rates during and after the disaster as evident from people in some clusters persisting with negative emotions even after the disaster due to a host of reasons. Our spatial analysis did not consider the social and demographic characteristics of the worst-affected clusters. Future studies need to take into account the social, demographic and environmental variables in their analysis so as to make in-depth, accurate and early predictions of the mental health status of the affected population.

In general, tweets expressing negative emotions dropped significantly after the disaster, leading us to conclude that display of high negative emotions was a temporary phenomenon during the disaster. Previous studies have shown that social connection and support, a simple act of kindness and sharing positivity result in the affected population turning to positive emotions [61, 62]. The post-disaster period in Chennai witnessed a huge community effort to reach out to the affected population, with volunteers from all walks of society rushing to help the worst-affected people, helping them cope, recover fast and build resilience [11, 63]. Instead of negative emotions, positive hashtags like *#ChennaiWeAreWithYou, #ChennaiRecovery, #RebuildingChennai* and *#BeingHuman* began to flood the Twitter feed. These positive actions might be one of the reasons for the significant decrease of negative thoughts among the affected population.

We also analysed the negative emotion tweets thematically to gain an insight into the issues that people considered as important during the disaster. If this data becomes available real time, it would help the relief teams and decision makers to plan response and recovery activities efficiently. The World Health Organization (WHO) stresses on providing psychological first aid, including emotional support, to the affected people during emergencies. The WHO recommends the including of at least one mental health professional in every general medical care relief camp organised during disasters [64]. We also call for the provision of mental health care assistance, especially after the disaster during the relief activities.

Our study has a few limitations. We considered geo-coded tweets in English and Tamil only, but there were people sharing their emotions in other languages also. We disregarded tweets that were not geo-tagged (not mentioning location). We are aware that communication was cut in several areas due to water logging and lack of power, preventing people in those areas from expressing their feelings and difficulties. So our study was not representative of the entire affected population. We used NRC lexicon method to classify emotions from the tweets. We are of the view that using domain-specific annotated training data for classification by machine learning techniques may offer a more efficient emotional classification [65, 66].

## Conclusions

The spatio-temporal analysis of emotions of the disaster-affected population using social media data helps to rapidly identify areas in which negative emotions run high so that areas needing immediate emotional support can be spotted early. If such spatio-temporal data can be generated in real time, it would help avoid the psychological fallout of the disaster by helping the relief teams to arrange for early mental health interventions.

## Supplementary information

**Additional file 1.** Geographical coordinates and ward number of negative tweets.

**Additional file 2.** Negative emotion tweet count and rates at ward level.

**Additional file 3.** Results of spatial regression model.

## Data Availability

The datasets analysed during the current study are not publicly available as the data involved are tweets from the public; ethically those cannot be published in the repository, but are available from the corresponding author on reasonable request.

## Abbreviations

API: Application programming interface
ANOVA: Analysis of variance
Tukey HSD: Tukey honest significant difference
AIC: Akaike info criterion
LISA: Local indicator of spatial association
WHO: World Health Organization
