# Supplementary material for: Spatio-temporal distribution of negative emotions on Twitter during floods in Chennai, India, in 2015: a post hoc analysis
Source: Int J Health Geogr. 2020 May 28;19:19. doi: 10.1186/s12942-020-00214-4 (PMC7254639; doi:10.1186/s12942-020-00214-4)
Supplement: Supplementary file 3 — Additional file 3. Results of spatial regression model. [file 12942_2020_214_MOESM3_ESM.docx]

**ADDITIONAL FILE 3 – Regression outcomes**

**SUMMARY OF OUTPUT: SPATIAL LAG MODEL - MAXIMUM LIKELIHOOD ESTIMATION**

**REGRESSION 1**

Dependent Variable - During disaster negative emotion rates

Independent Variable - Pre disaster negative emotion rates

**Model Diagnostic**

R-squared 0.626287

Log likelihood 159.531

Akaike info criterion -313.062

Sigma-square 0.00303755

S.E of regression 0.055114

**Spatial dependence diagnostic**

Likelihood Ratio Test

Value 86.9539

Probability 0.00000

**Outcome**

| **Variable** | **Coefficient** | **Std.Error** | **z-value** | **Probability** |
| --- | --- | --- | --- | --- |
| Constant | 0.0868465 | 0.0208272 | 4.16986 | 0.00003 |
| Negative emotion rate (Pre disaster) | 0.0134352 | 0.0689161 | 0.194951 | 0.84543 |

**REGRESSION 2**

Dependent Variable - Post disaster negative emotion rates

Independent Variable - Pre disaster negative emotion rates and

During disaster negative emotion rates

**Model Diagnostic**

R-squared 0.453046

Log likelihood 193.343

Akaike info criterion -378.687

Sigma-square 0.00134018

S.E of regression 0.0366085

**Spatial dependence diagnostic**

Likelihood Ratio Test

Value 41.1405

Probability 0.00000

**Outcome**

| **Variable** | **Coefficient** | **Std.Error** | **z-value** | **Probability** |
| --- | --- | --- | --- | --- |
| Constant | 0.0250268 | 0.0144918 | 1.72696 | 0.08417 |
| Negative emotion rate (Pre-disaster) | -0.0934274 | 0.0501417 | -1.86327 | 0.06242 |
| Negative emotion rate (During-disaster) | 0.0985898 | 0.0432804 | 2.27793 | 0.02273 |
